# Dynamics of optical properties of sequentially diluted lucigenin aqueous solutions according to luminescence data

**DOI:** 10.3389/fchem.2024.1439250

**Published:** 2024-10-16

**Authors:** Dmitrii L. Tytik, Viktor I. Kuzmin, Olga V. Souvorova, Alexandra A. Revina

**Affiliations:** ^1^ Frumkin Institute of Physical Chemistry and Electrochemistry Russian Academy of Sciences (IPCE RAS), Moscow, Russia; ^2^ Russian Technological University (MIREA), Moscow, Russia

**Keywords:** spectrofluorophotometry, aqueous solutions of lucigenin, sequential ultra-high dilutions, luminescence, relaxation

## Abstract

The article discusses optical properties (luminescence) of diluted (24 dilution factor) lucigenin (*Lc*) aqueous solutions. Six series of *Lc* aqueous solutions, with 50 samples in each series, were studied. The series were diluted on different days within random schedules, following a unified procedure: the first sample in all the series was the *Lc* (C_*Lc*_ = 8.2 × 10^−7^ mol/L) stock solution, while the rest of the samples were obtained by successive dilution with the ratio of 24. For the first three samples, the *Lc* luminescence intensity decrease appropriately complied with the exponential function model (the dilution ratio: none for the stock solution, for the second and the third, 24 and 24 × 24 = 24^2^, respectively). Starting from the fourth sample for statistical processing of luminescence data, the seven largest values were selected from the built rank distribution of emission intensity values. This method helps eliminate the influence of “random large bounces” when calculating the correlation coefficient. Up to the 50th studied sample, a challenging linear gradual decrease in the intensity of recorded photometric values was noted (correlation coefficients for all series being close to −0.9). Similar analysis of six reference series of pure water “dilution” samples did not exhibit any correlation between the highest emission values in the studied wavelength range (specific for *Lc* bandwidth, 480–505 nm) and the sample’s dilution number. It can be assumed that photometric values, recorded in the series of Lc sequentially diluted aqueous solutions after substance (*Lc*) elimination (theoretically expected after the 13th sample within the used experimental setup), could be attributed to the gradual destruction of long-lived aqueous structures formed in the process of hydration of *Lc* molecules during its dissolution.

## 1 Introduction

At present, two types of dilution (mixing) are mainly used in the preparation of aqueous solutions: continuous (by D. I. Mendeleev), when solute concentration increases/decreases gradually, and discrete, with a sharp change in the concentration (sometimes by tens and hundreds of times in the dilution ratio). The founders of the first direction, D. I. Mendeleev and N. S. Kurnakov, laid the experimental and theoretical foundations for the studies of physicochemical properties of aqueous solutions. At the same time, D. I. Mendeleev was apparently the first to look at solutions not as a mechanical mixture of components but as special chemical compounds after he had discovered the phenomenon of changes in the specific volume (compression) of aqueous solutions of alcohol with an increase in its concentration accompanied by the appearance of breaks and jumps in the solution density derivatives with respect to concentration (Mendeleev and Lawson, 2016). D. I. Mendeleev associated this property with the formation of hydrated compounds in a solution in the vicinity of the corresponding alcohol concentrations. The second direction (discrete dilution/sequential dilution) existing from approximately the end of the 18th century and aimed at obtaining aqueous solutions of ultra-low concentrations caused heated discussions about the reality of the declared effects. Perhaps, at the end of the 20th century, the most resonant research was studying by natural science methods of verification of changes in the physicochemical properties of aqueous solutions with a substance in an ultra-low concentration, the results of which were published in a Nature journal article in 1988 (Davenas et al., 1988). A supposition was made that the observed effects may be caused by the peculiarities of the preparation of aqueous solutions of ultra-low concentrations, involving vigorous shaking, thus leading to the appearance of long-lived molecular structures of water. Consideration of the effects of preparations based on ultra-high dilutions on biological models has become essential for research performed these days not only at a molecular level but also at a supramolecular level, including studies of a living body and its functions (Epstein, 2018; Tarasov et al., 2020). At the same time, not only the specifics of the dilution process but the dilution ratio itself, selected for the preparation of ultra-high dilutions^1^, which, according to different authors, should have definite selected values, might determine the result (Tarasov et al., 2020; Smirnov, 1908). In the case study by Revina et al. (2020), when lucigenin (*Lc*) was dissolved in water and further multiply diluted, the presence of certain rhythmic patterns was shown.

One of the conditions for the preparation of highly diluted aqueous solutions is, as a rule, a high dilution ratio at each iteration step. Theoretical estimation, if made taking into account Lc’s initial concentration C = 8 × 10^−7^ mol/L (as in the work described in this article), which corresponds to 5 × 10^14^ *Lc* molecules in 1 mL of water (sample no. 1), and dilution rate of 24, suggests entire elimination of *Lc* molecules from the solution by the 13th successive dilution, and, consequently, provokes questions related to the applicability of analytical methods and instrumentation used in research.

Recently, several remarkable achievements were made in the quest for reliable measurements at extremely low concentrations via the path of improving high-sensitivity and high-selectivity of spectrofluorophotometric instruments. Duloxetine hydrochloride detection with 0.0002 μg/mL (0.2 ppb) lower limit and 0.0007 μg/mL (0.7 ppb) lower limit of quantitation has been obtained by fluorescence spectroscopy by Maruyama and Fuyji (2019). No less persuasive examples of high-sensitivity analysis of fluorescein claiming fluorescence detected on the order of 1 × 10^−13^ mol/L (sub-picomol) compared with a blank sample (purified water) have been reported by Jun and Iwaya (2017). Some instruments, such as Hitachi fluorescence spectrophotometer F-7000 used for recoding the emission intensity from c.a. 8,000 to less than 8 a.u. range, have the automatic gain change-over function implemented, which is a technique that makes it possible to generate calibration curves using up to 6-digit values and measuring unknown samples without additional sample preparation.

Along with instrumental advances, further research, designed for studying experimental samples with concentrations “beyond determination rate,” might benefit from approaches that step aside from non-productive paths of disputes regarding quantification of the substance at ultra-low concentrations beyond ultra-trace levels. For example, it might be suggested to rearrange the “substance–solvent” focus of the study from the quantity of dissolved *substance* to the changes in the physicochemical properties of the *solvent* itself (water) in the process of multiple repeated substance dilution procedures and to the conditions of the mechanical activation applied.

With a continuous dilution of solutions, the solvent performs an auxiliary function when the process of dissolving the substances causes the occurrence of chemical reactions to be impossible, for example, in a solid state. Until some time ago, less attention was paid to the issues of transformation of the solvent itself (water). For example, changes in the solvent structure under the impact of a solute have not been sufficiently studied, and such studies became possible only with the development of numerical experiment methods (for example, molecular dynamics (Allen and Tildesley, 2017)).

Currently, many anomalous properties of water are associated with the presence and properties of the network of hydrogen bonds, their dynamics (Geiger and Stanley, 1982), formation of nanobubbles of the dissolved gases (Bunkin et al., 1995; Bunkin et al., 2019; Tytik et al., 2019), the processes of hydration of the solute molecules, and many other factors. The use of numerical methods made it possible to study dynamics of the molecular structure of complex micro-heterogeneous aqueous systems, using, for example, the concept of a random network of hydrogen bonds (Geiger and Stanley, 1982; Rodnikova, 1993; Rodnikova and Barthel, 2007). Therefore, when analyzing the effects observed in highly diluted aqueous solutions, it is necessary not only to take into account modern ideas about the water molecular structure and the role of hydrogen bonds but also to use them for building the models for interpretation of anomalous properties of solutions. The aim of the work was the confirmation or refutation of the presence of physicochemical effects caused by sequential dilution (with a sufficiently large ratio of 24) of aqueous solutions of lucigenin applying fluorophotometry as a high-resolution optical analysis method and utilizing high-sensitivity sensors^2^. Fluorophotometric measurement provides multicomponent signal data, comprising both the specimen and solvent luminescence, combined with multiple light-scattering effects (due to Raman scattering, reflectance from chamber side walls, gas nanobubbles present in the solution, and liquid density fluctuations). As the substance concentration decreases after each aqueous dilution, there comes a point when its characteristic luminescence peak is disguised with background water molecular light scattering. To lessen concerns regarding the instrument’s sensitivity and substance determination levels, becoming most prominent when low signals, approaching the noise level, are being considered, the control measurements (blank sample of water used for dilution, water after its vigorous shaking within “pseudo dilution” process, signal record with an empty cell, and that from an empty chamber) were made in similar conditions to verify that the intensity data taken for analysis exceeded the blank and control values. The task was to record and not miss whatever change in optical properties might have taken place during the *Lc* dilution process. The article contains an analysis of the luminescence spectra of six series of aqueous solutions (50 samples each) obtained by sequential dilution of the stock solution of *Lc* (sample No. 1) and the preparation of 49 more sequential dilutions (samples) in each series (sequential dilution with the ratio of 24). Mathematical processing of luminescence spectra of the *Lc* dilution series in the range of 480–505 nm was carried out and studied in comparison with the data recorded for six series of aqueous water “pseudo-dilutions” prepared in accordance with a similar sequential procedure, with the only difference being the total absence of *Lc* in sample No. 1 as pure stock water was used instead.

## 2 Materials and methods

**FIGURE 1:**
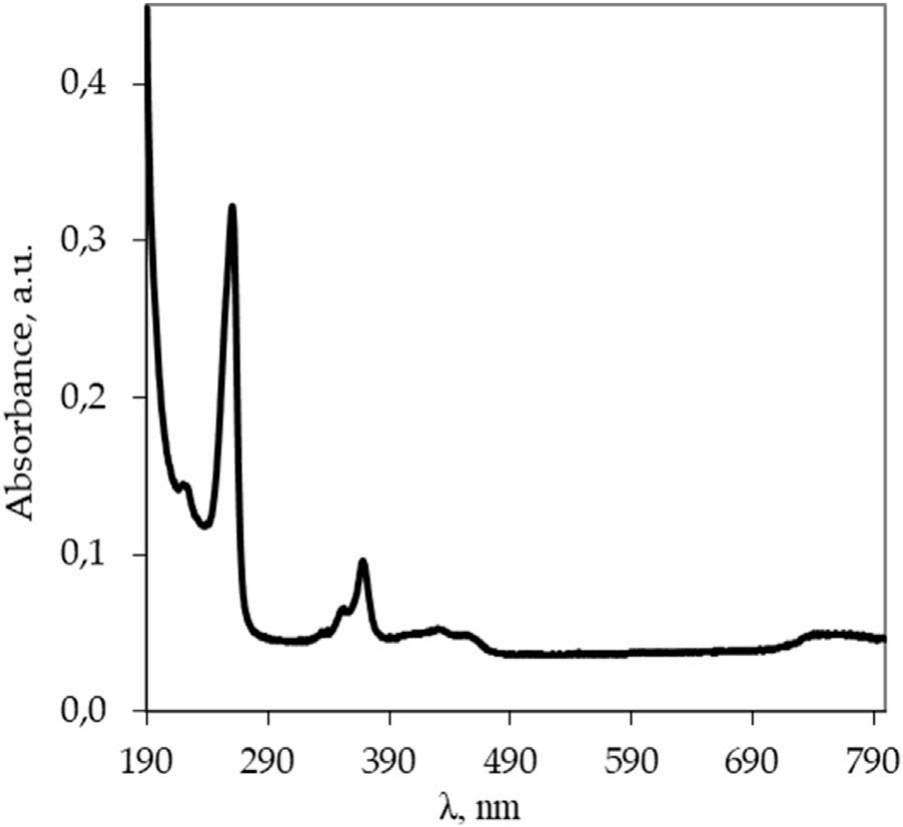
Optical absorption spectrum of the *Lc* aqueous solution (C_*Lc*_ = 0.7 mg/L) recorded in a quartz cell (path length 10 mm) and Milli-Q water in quartz cell as a blank sample using a UV-vis spectrophotometer U-3310 (Hitachi).

To prepare all the aqueous solutions, including aqueous solutions of *Lc* ^3^, we used the water prepared in the Millipore system (cleaning class 1, electrical resistivity σ = 18 MΩ cm). First, an aqueous solution of the substance (C_*Lc*_ = 0.7 mg/L) was prepared to record the optical absorption spectrum (OAS, U-3310, Hitachi) and select the wavelength for luminescence excitation (F-7000, Hitachi) in aqueous solutions of *Lc*. A quartz cuvette with the optical path length of 10 mm was used; the OAS was recorded relative to water. Figure 1 shows the OAS for an aqueous solution with the concentration of *Lc* (C_*Lc*_ = 0.7 mg/L). The OAS clearly shows two light absorption maxima at wavelengths λ = 260 and 367 nm, and wavelength λ = 260 nm was chosen as the excitation wavelength for the luminescence measurements of *Lc* aqueous diluted samples as its intensity was higher than the intensity during excitation at 367 nm.

The following fixed dilution parameters were used in the preparation of aqueous solutions of *Lc*:

1) The volume of aqueous solutions remained unchanged at each dilution (*V*_*i*_ = 24 mL).
2) To prepare the next solution (iteration i), a part of the solution (*V* ^*p*^_*i*-1_ = 1 mL) of the previous dilution (iteration *i* - 1) was used so that for each dilution, the condition (*V*_*i*_ */V* ^*p*^_*i*-1_ = *K*, where *K* is the dilution ratio) was observed.

In this case, the *i*-th dilution ratio is calculated by the formula $K_{i}=K^{\;i-1}$ (where i≥2 , and *K* is the dilution ratio at the *i*-th iteration step with respect to the first solution). It should be noted that this formula does not primarily relate to the solute concentration; it is just a convenient record of the procedure for sequential dilution of this substance. At each iteration, the solution after dilution was subjected to mechanical activation (stirring/shaking) according to the Hahnemann method. So, in this work, the dilution ratio of 24 is chosen; therefore, as an example, the 10th solution has the dilution ratio $K_{10}=24\times 24\times ...\times 24=24^{\;9}$ with respect to the first (stock) solution.

The *Lc* concentration in the original sample No. 1 was selected so that the maximum intensity of the luminescence range of this sample would not go beyond the detection limit of the fluorimeter (Hitachi F7000). Thus, the *Lc* initial concentration in sample No. 1 with the volume of 24 mL was chosen, which was C_*Lc*_ = 8.2 × 10^−7^ mol/L. This solution was used as the stock solution for the preparation of the remaining 49 samples of the *Lc* series.

Each sequential dilution (i.e., preparation of each sample) was carried out in an individual clear borosilicate glass vial (GTG Glastechnik Grafenroda, GmbH, Germany, V = 40 mL), according to the following procedure.

**FIGURE 2:**
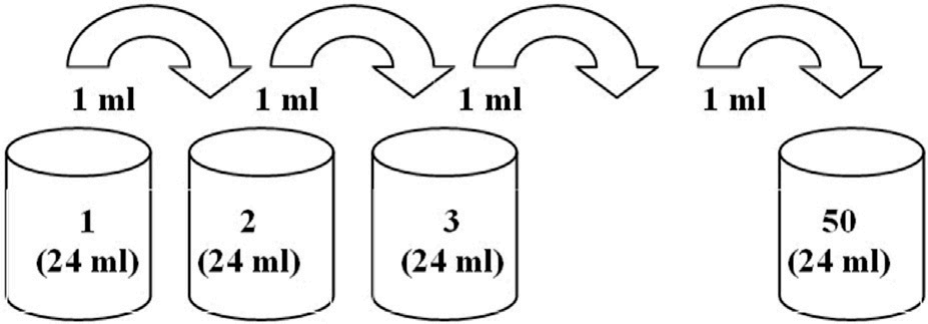
Sequential dilution procedure scheme.

1) *Lc* stock solution (No. 1, C_*Lc*_ = 8.2 × 10^−7^ mol/L) with the volume of 24 mL was placed in a 40-mL glass vial.
2) The second solution (dilution No. 2) was obtained according to the following scheme: 12 mL of Millipore water was poured into an empty vial, 1 mL of the *Lc* stock solution (sample No. 1) was added, and then 11 mL of Millipore water was added to the total volume of 24 mL, after which the vial with the resulting solution was manually shaken (21 strokes, 20 cm amplitude, 310 g impact force, with the starting point of sample location at 21 cm height/distance from the lab buffer cushion) at the frequency of approximately 4 Hz for approximately 5 s. The described procedure corresponds to the dilution ratio of 24.
3) All subsequent dilutions, Nos 3–50, were carried out in a similar way according to the scheme (Figure 2) by adding 1 mL of the previous dilution. A pipettor with a plastic tip was used to withdraw 1 mL of the solution, which was replaced with a new tip at each dilution iteration. The solution volume (1 mL) was taken approximately from the middle of the solution volume (24 mL). The newly prepared solution (24 mL) was placed in a glass vial (40 mL) with a plastic screw cap^4^.

Wavelength scans of all samples, including the blank, with measurement sessions starting with the 50th sample in all the series (from 50th to 1st, i.e., from “zero” to stock *Lc* concentration “increase” logic), in the range of 200–900 nm with 260-nm excitation bandwidth, were run in the emission scan mode at a speed of 240 nm/min, excitation and emission bandwidths set at 5 and 2.5 nm, respectively, and the PMT voltage set at 700 V.

**FIGURE 3:**
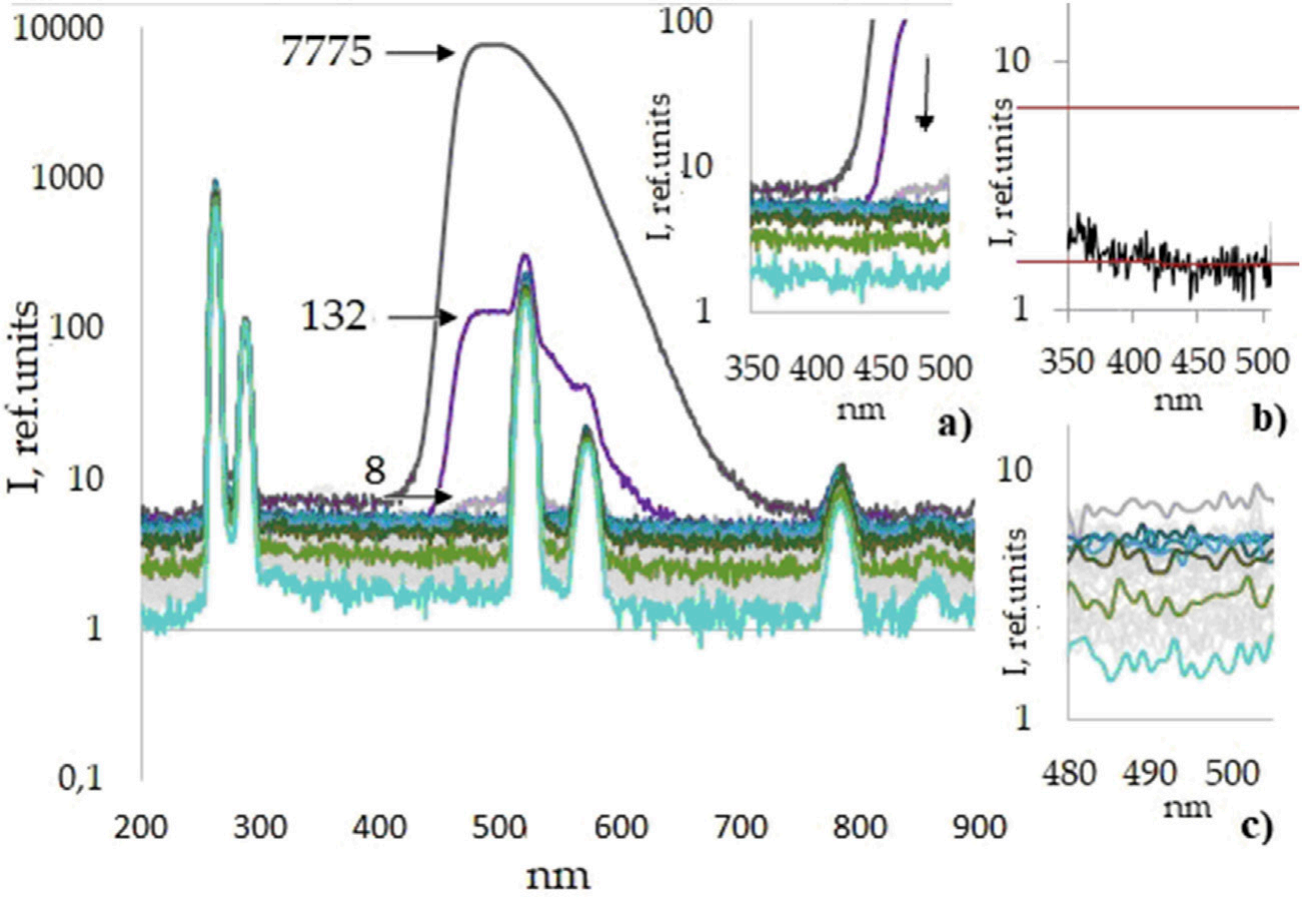
Luminescence spectra of the sixth series of *Lc* aqueous solutions/sequential dilutions (Nos. 1–50), including a blank, semi-logarithmic scale. For the first three samples (*Lc* stock solution and dilution nos 2–3), black arrows indicate the values of the maxima of the *Lc* luminescence intensity. Excerpts: selected spectra in 350–500 nm range, nos 1–4, 10, 20, 30, 40, and 50: 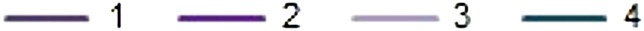 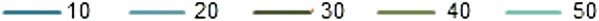 **(A)**; blank sample (water), red horizontal lines indicate intensity levels of sample No. 3 (upper line) and blank sample (lower line) **(B)**; intensity values of all samples within the sixth series (Nos: 1–50) in the 480–505 nm range selected for rank distribution assessment **(C)**.

Figure 3 shows the luminescence spectra of the sixth series of aqueous solutions of *Lc* (50 dilutions/samples). The luminescence spectra of the five other series are similar. Figure 3 shows the luminescence spectra with well-developed Rayleigh elastic scattering bands caused by excitation of the samples at wavelength λ = 260 nm, the main line at 260 nm and its two harmonics with a lower intensity (λ = 520 and 780 nm), and Raman scattering bands (λ = 287, 574, and 861 nm) near them. The Raman shift is calculated by the formula Δν = (1/λ_0_ – 1/λ_1_), is approximately 3,600 cm^−1^, and corresponds to the stretching vibrations of the OH bond in the water molecule. It should be noted that the intensity of elastic scattering bands qualitatively characterizes the presence of scattering centers in aqueous solutions; for example, the nanobubbles of atmospheric gases of different sizes, which were shown in Bunkin et al. (1995), Bunkin et al. (2019), and Tytik et al. (2019). Figure 3 shows a rapid initial decrease in the luminescence intensity corresponding to the lucigenin band (480–505 nm), which can be described by the exponential function. A small peak (c.a. 8 a.u.) in the *Lc*-specific range is prominent in the spectrum of sample No 3. In a 24-mL solution of this sample, c.a. 10^13^–10^14^ *Lc* molecules can be contained, judging by the stock solution concentration and used dilution rate. This estimated concentration can be regarded as the *Lc* determination limit within the frame of the conducted experiment as at this stage, F-7000 sensitivity enables recoding *Lc* molecules' presence. Starting from the fourth sample, the luminescence intensity of the aqueous solution in the wavelength range of the *Lc* band significantly decreases and remains in the lower intensity range, which indicates a decrease in the number of *Lc* molecules, leading to the assumption that the *Lc* molecule number has reached ultra-trace levels or that *Lc* is already absent. The difference between the spectra of the 10th, 20th, and 30th samples, shown for clarity in excerpt Figure 3A, is rather small, although distinguishable from those of the 40th and final 50th samples seen below them. At the same time, photometric values in the *Lc*-characteristic studied the luminescence range remain above the level of the blank sample (Figure 3B) up to the 49th studied sample and are disguised with the background water molecular light scattering.

## 3 Results and discussion

The following filter was developed for mathematical processing of the luminescence spectra of *Lc* aqueous sequential dilutions. For making analysis, we selected a narrow band of the luminescence spectrum in the wavelength range 480–505 nm (Figure 3C), which corresponds to the *Lc* luminescence band in the initial sample. The choice of the right end of the range (wavelength 505 nm) was determined to completely exclude any impact of the band of the second harmonic of elastic scattering, with the maximum at 520 nm. The intensity of this band in the *Lc* luminescence spectrum of the stock solution starts to increase from the wavelength of 506 nm. The left end of the range with the wavelength of 480 nm was selected so that part of the luminescence spectrum of the stock aqueous solution of *Lc* (No. 1) had an approximately symmetrical shape relative to the middle of the selected range. So, the built filter was a criterion for data selection, followed by a subsequent analysis of the luminescence spectra of the aqueous solutions of all dilutions.

The luminescence spectra were recorded with 1-nm discreteness; thus, there are 25 luminescence intensity values in the range of 480–505 nm (sample). For each such sample of 50 spectra, a rank distribution was built, i.e., the luminescence intensity values were ordered (ranked) in an ascending order. To compensate random large “outliers” of the luminescence intensity values in the selected range, the seven largest values were selected from the ranked sample. At the final stage of data preparation, an array of 47 groups of seven intensity values from each luminescence spectrum of aqueous solutions (Nos. 4–50) was formed. Sample nos 1–3 were not included in the analysis at this stage as the luminescence intensity values for them are 1–3 orders of magnitude higher than those for highly diluted aqueous solutions.

**FIGURE 4:**
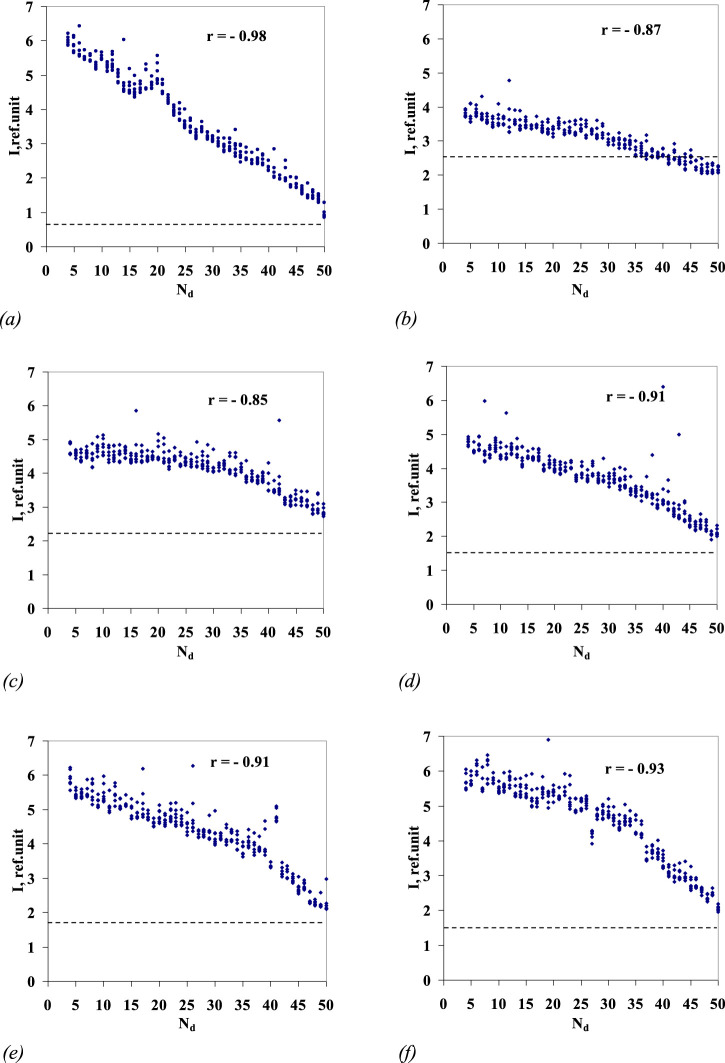
Highest values of luminescence intensity in the wavelength range of 480–505 nm (*y*-axis) for series 1–6 (**(A–F),** respectively) of *Lc* aqueous solutions/sequential dilutions No. 4–50 vs. sample number (N_d_ dilution number) (*x*-axis). For the series of *Lc* aqueous solutions/sequential dilutions nos 1–6, the correlation coefficient *r* is given. The dotted line marks the level of luminescence intensity of the blank sample, the stock water used for preparation of samples nos 1–50. The values of the luminescence maxima for samples nos 1–3 of the sixth *Lc* dilution series are shown in Figure 3 by arrows.

At the final stage of processing, all six series of the samples represented by arrays of 329 (= 47 × 7) luminescence intensity values in each, associated with the sample number, were ranked by intensity values (rank distribution). Figure 4 shows the rank processing results. It should be noted that a correlation of the aqueous solution luminescence intensity with the sample number is found (the correlation coefficients are close to −0.9) for all the series of aqueous solutions.

A trial shift of the range (“window”) for the spectrum-processing filter was performed; for example, in the range 370–395 nm, in which the *Lc* band is absent, the correlation between the luminescence intensity and the sample number is preserved, and the correlation coefficients do not actually differ from the correlation coefficients calculated for the range 480–505 nm.

**FIGURE 5:**
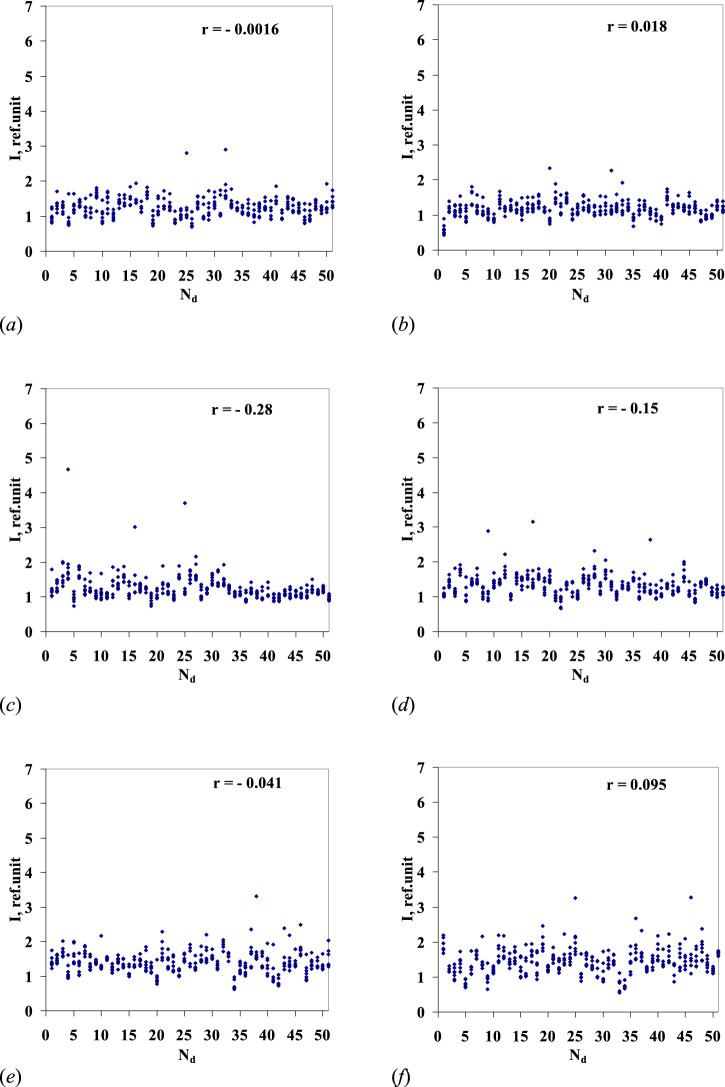
Highest values of luminescence intensity in the wavelength range of 480–505 nm for six series of 50 sequential “pseudo-dilutions” of water (*y*-axis) vs. the dilution number Nd (*x*-axis). For series nos 1–6 (**(A–F)** – respectively), correlation coefficient r is given. Sample No. 51 corresponds to the source water (blank) in each of the six series.

Figure 5 shows the results of similar mathematical processing of luminescence spectra of the water samples (Nos. 1–50) diluted according to the above procedure, with the ratio of 24, followed by mechanical activation. All samples (dilution Nos 1–50) together with sample No. 51 (blank/initial water) in each series were included in single analytical processing. In this case, for all six series of “pseudo-dilutions” of water, a correlation is almost non-existent between the solution luminescence intensity and the solution’s dilution number.

**FIGURE 6:**
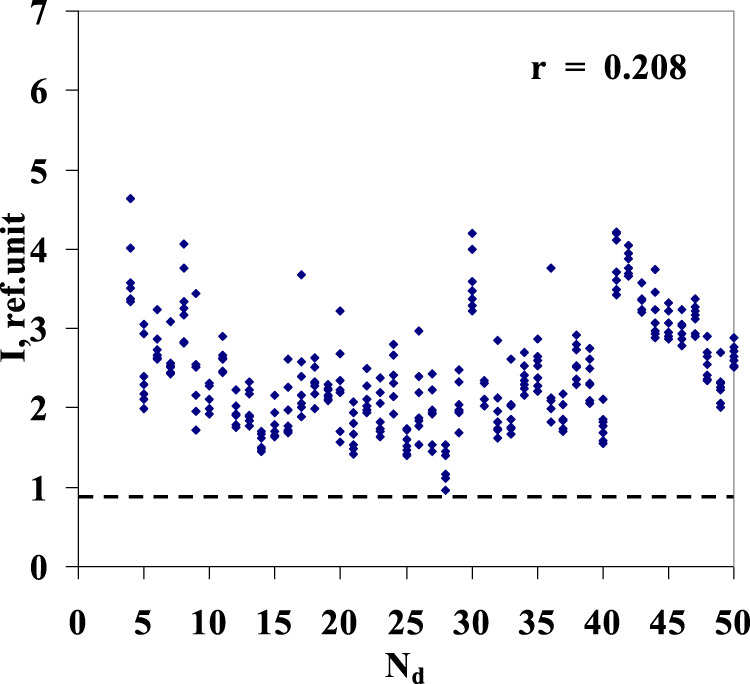
Highest values of luminescence intensity in the wavelength range of 480–505 nm of the fourth series of *Lc* aqueous solutions/sequential dilutions (Nos. 4–50) recorded after the 7-month storage term. The dotted line indicates the level of luminescence intensity of the stored blank sample of the fourth series, the initial water used for preparation of *Lc* dilutions.

Reported optical properties of the *Lc* aqueous dilutions change with time and gradually vanish without a trace, as illustrated in Figure 6. After 7 months, the results of luminescence measurements of diluted *Lc* samples are virtually undistinguishable from one of six control series of pure water sequential “pseudo-dilutions” (Figure 5).

Thus, in the process of sequential dilution, the effect of *Lc* aqueous solution relaxation was found. It manifests itself as a linear decay of luminescence intensity values in the course of the sequential dilution of the solution beyond sample No. 4 all the way through up to sample No. 50 (for which the dilution ratio with respect to the stock solution is $K_{50}=24\times 24\times ...\times 24=24^{49}$ ).

It should be noted that usually, relaxation (lag) characterizes the integral response of the system. The observed relaxation phenomenon is understood to be caused by both external excitation (in a form of the mechanical activation adopted for the dilution technique) and *Lc* presence at the starting point of sample preparation in the stock solution. The latter conditionality is supported by the results of comparison made with six reference series of experimental “pseudo-dilutions” of pure water, performed in accordance with a similar procedure, when recorded luminescence data demonstrated no correlation between the luminescence intensity of the solution (the sample) and the sample number (Figure 5). The noted effect may relate to the peculiarity of *Lc* dissolution in water: although the mechanical activation procedure remained the same during all performed experiments, the emission decline trend got registered only in case the water had “contact” with *Lc*.

One cannot help mentioning a common concern that repeated accomplishment of dilution procedures might cause a certain amount of the solute’s molecules to be floating in the upper layers of the solutions and/or migrating from one vial to the next one during the dilution process due to absorption on plastic pipette tips and/or as a result of capture in the water–gas (bubbles) interfacial region with further flotation, as in Bunkin et al. (2019). The quantity of such immigrant molecules is scarce and deemed to be less than the minimal number sufficient for valid registration using standard fluorometric methods of the concentration determination. Nevertheless, when/if present, they must be involved in stabilization of the heterostructures in the solutions, thus participating in the transformation of the physical properties of the solutions at various stages of the dilution procedure. It should be noted, however, that the methods used in the present study are not useful for the control of the trace substance concentrations (for example, beyond 10^−10^ -10^−13^ M), and, as such, the “migration” factor could not have been technically assessed. Thus, for the characterization of evidently high dilutions ( $K_{50}=24\times 24\times ...\times 24=24^{49}$ ), it seems appropriate to discuss the behavior of the solvent (water) after its contact with the solute (*Lc*) at the first stages of the sequential dilution process, rather than to look for traces of its presence in the aqueous solution at the *i*-th iteration step, beyond the present substance determination capabilities. Apparently, during the *Lc* dissolution in water and at further dilution in the process of mechanical activation (stirring/shaking), a significant transformation of the hydrogen bonding network occurs, causing formation of such long-lived structures as nanobubbles of atmospheric gases (Bunkin et al., 1995; Gudkov et al., 2020), reactive oxygen species (Gudkov et al., 2020; Gudkov et al., 2021), or/and stable clusters. Appearance of the interfacial region of nanobubbles is also an additional factor having an impact on the *Lc* dissolution (adsorption in the interfacial region). In other words, complex physical processes occurring in a solution with multiple successive dilutions, accompanied by an intense mechanical activation effect, make it possible to obtain solutions with new physicochemical properties that might have been conditioned by the composition of the stock solution, but at certain dilution stages, they are no longer determined by the actual physical presence of molecules of the original substance in them.

Presently, within the solvent-shifted paradigm the photometric experiments outlined in this study, we provide evidence of the presence of scattering centers in an aqueous solution marked by elastic scattering bands (the main excitation line λ = 260 nm and its harmonics). Although nanobubbles are reliably registered by a number of methods, phase microscopy, DLS, and small-angle neutron scattering, the identification of stable *Lc*–*Lc*, *Lc*–water, and water–water clusters is still far from being successfully accomplished by the majority of modern high-resolution instruments. Promising results were described in Penkov and Fesenko (2020) and Penkov (2019) when a new low-energy THz-spectroscopy approach was used for determining the morphological changes in hydrated supramolecular biological and non-organic structures, suggesting the THz-method’s good potential for further investigation of the transformations of hydrogen bonds and substance–water, water–water, and water–gas interfacial areas caused by intense mechanical stress and applied dilution techniques.

## 4 Conclusion

In the process of sequential dilution of the aqueous *Lc* solution, it was found that the *Lc* concentration and luminescence intensity of the first three samples (Figure 3) decreased according to the exponential law, which corresponds to the chain monomolecular chemical reaction model and relaxation process discussed in some earlier studies (Kuz’min et al., 2015a; Kuz’min et al., 2015b; Kuz’min et al., 2015c). In the vicinity of the fourth dilution, the trend had changed as the luminescence intensity decay became almost linear, depending on the dilution number. The observed phenomena were recorded in six separately run *Lc* dilution series. Reference experiments with water, during which the sequential dilution procedure was fully replicated up to the same level of “high dilutions,” showed no such correlation (between the luminescence intensity and the dilution number).

In the present study, we registered the manifestation of the photometric declining trend characteristic for sequentially diluted samples of *Lc* dissolved in water in the vicinity of the dilution range when the substance, in accordance with estimates, must have been entirely eliminated. It should be noted that the phenomena of the luminescence intensity decay (continuing after *Lc* 13th dilution) in the wavelength range of 300–500 nm (Figure 3) can be interpreted as a decrease in molecular scattering. In this case, molecular scattering should be understood in the generally accepted sense as scattering due to liquid density fluctuations caused by a change in the properties of the solvent during the dilution process. This effect can be attributed primarily to the water (solvent) relaxation properties at the level of possible formation of long-lived stable hydrate molecular structures. Determination of the specific contribution of multiple components (luminescence, light scattering, etc.) in the scanned spectra of aqueous *Lc* diluted solutions in the substance-specific range represents a stand-alone problem, and it requires further research with another experimental set-up.

The stability boundaries and possible molecular models (Lebedev et al., 2021; Penkov, 2019) of these structures and the mechanism responsible for their transformation (Lobyshev, 2023) are expected to be investigated in further studies with an extended range of soluted substances.

## Data Availability

The luminescence intensity datasets of six series of luigenin aqueous solutions (24 dilution factor), 50 samples in each series, is available on request. Requests to access the datasets should be directed to Dmitrii L. Tytik, dtytik@yandex.ru.

## Footnotes

1 By definition, ultra-high dilutions are obtained from the initial aqueous solution of a substance by choosing a certain (sometimes tens and hundreds) dilution ratio, which remains constant in successive dilutions.

2 Fluorescence Spectrophotometer Hitachi F-7000: specified sensitivity (signal-to-noise ratio): S/N 800 or above (RMS) upon measuring Raman spectrum of water, S/N 250 peak to peak. F-7000 serial number 1910-020 random laboratory check record: S/N 906 (RMS) upon measuring Raman spectrum of water, S/N 312 peak to peak, noise levels: N peak to peak 0.337, N RMS 0.116.

3 Lucigenin (10,10′-dimethyl-9,9′-biacridinium dinitrate, *Lc*), molecular weight 512.51; golden yellow crystals; melting point 330°C; soluble in water, poorly soluble in ethanol.

4 Control of the uniformity of the diluted substance’s distribution in the volume of the sample, which theoretically might be impacted by the solute’s uneven concentration at liquid-to-solid and liquid-to-air interfaces, or particles flotation on the surface, as suggested in Allen and Tildesley (2017), was beyond the goals of the present study due to the experiment’s design. Possible non-uniformity was not taken into account, as main studies of the solvent were undertaken in the range far beyond the point at which molecules of the solute are usually expected to be present or detectable.
