# Supplementary figures and images for: Dynamics of optical properties of sequentially diluted lucigenin aqueous solutions according to luminescence data
Source: Front Chem. 2024 Oct 16;12:1439250. doi: 10.3389/fchem.2024.1439250 (PMC11521942; doi:10.3389/fchem.2024.1439250)

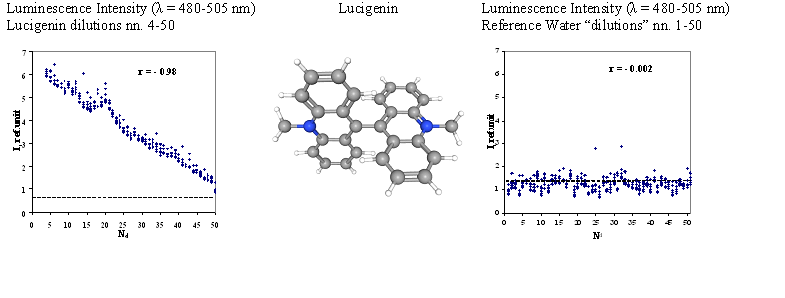

Supplement: Supplementary file 1 [file Image1.TIF]
